# Diatom bloom-derived biotoxins cause aberrant development and gene expression in the appendicularian chordate *Oikopleura dioica*

**DOI:** 10.1038/s42003-018-0127-2

**Published:** 2018-08-24

**Authors:** Nuria P. Torres-Águila, Josep Martí-Solans, Alfonso Ferrández-Roldán, Alba Almazán, Vittoria Roncalli, Salvatore D’Aniello, Giovanna Romano, Anna Palumbo, Ricard Albalat, Cristian Cañestro

**Affiliations:** 1 Departament de Genètica, Microbiologia i Estadística and Institut de Recerca de la Biodiversitat (IRBio), Facultat de Biologia, Universitat de Barcelona. Av. Diagonal 643, 08028 Barcelona, Catalonia Spain; 2 Department of Biology and Evolution of Marine Organisms, Stazione Zoologica Anton Dohrn, Villa Comunale 80121, Napoli, Italy; 3 Department of Integrative Marine Ecology, Stazione Zoologica Anton Dohrn, Villa Comunale, 80121 Napoli, Italy

## Abstract

Investigating environmental hazards than could affect appendicularians is of prime ecological interest because they are among the most abundant components of the mesozooplankton. This work shows that embryo development of the appendicularian *Oikopleura dioica* is compromised by diatom bloom-derived biotoxins, even at concentrations in the same range as those measured after blooms. Developmental gene expression analysis of biotoxin-treated embryos uncovers an aberrant golf ball-like phenotype affecting morphogenesis, midline convergence, and tail elongation. Biotoxins induce a rapid upregulation of defensome genes, and considerable delay and silencing of zygotic transcription of developmental genes. Upon a possible future intensification of blooms associated with ocean warming and acidification, our work puts an alert on the potential impact that an increase of biotoxins may have on marine food webs, and points to defensome genes as molecular biosensors that marine ecologists could use to monitor the genetic stress of natural populations exposed to microalgal blooms.

## Abstract (web summary)

Torres-Águila et al. report that embryo development of the appendicularian chordate *Oikopleura dioica* is affected by biotoxins released by phytoplankton blooms. This work shows that these biotoxins may impact marine food webs and that defensome genes of appendicularians could be used to monitor the genetic stress of natural populations.

## Introduction

Climate change impact on ecosystems is complex and often with no direct relationship. Nevertheless, it is conceivable that pressures such as ocean warming and acidification may potentially intensify the severity and frequency of harmful algal blooms, globally influencing marine planktonic systems^1^. Harmful algal blooms include diatoms, which are among the most important photoautotrophic organisms that drive marine food web dynamics^2^. Diatoms can produce different biotoxins, some of which may act as a defense mechanism against grazers^3–6^. Among diatom-derived biotoxins, oxylipins are of prime interest because of their negative effects on the reproduction of copepods^4^, the main grazers of these algae and one of the most abundant components of the mesozooplankton^7^. Oxylipins, which include polyunsaturated aldehydes (PUAs), are secondary metabolic end-products of a lipoxygenase/hydroperoxide lyase pathway that are toxic when released to the environment^8^. During algal blooms, cell membranes are broken by cellular friction, massive grazing or senescence at the end of the bloom, generating oxylipin-rich microzones that can alter the biology of neighboring organisms^9^.

Oxylipin toxicity does not only affect copepods, but recent studies have shown that several marine species are compromised, including sea urchins^9–13^, ascidian urochordates^14–16^, as well as mollusks and annelids^10,17,18^. Despite the fact that analyses of different organisms have shown that PUA’s toxicity can affect a wide range of physiological processes, including oocyte maturation, sperm motility, fertilization, cell proliferation, embryogenesis, hatching, metamorphosis and apoptosis (reviewed in the ref. ^19^), the molecular bases of PUA’s toxicity remain often unclear. Studies in copepods and ascidians have revealed, for instance, that PUAs affect the expression of stress response genes (i.e., defensome^20^) associated with the metabolism of the glutathione system (e.g., *Gclm*, *Ggt*, and *Gst*)^15,16,21,22^. The expression of defensome genes related to aldehyde detoxification derived from lipid peroxidation (e.g., *Aldh2* and *Aldh8*) are also altered by PUAs, suggesting that these compounds may also induce oxidative stress^21,23,24^.

While the physiological effects of PUA’s toxicity have been investigated, the developmental genetic mechanisms affected by PUAs causing embryo malformations remain largely unknown. In sea urchins and ascidians, expression analyses by qRT-PCR of developmental genes related to embryo patterning (e.g., *Hox*, *Parahox*) or signaling pathways (e.g., *Wnt* and *nitric oxide/Erk*) have revealed differences in the expression levels^15,16,24^. The mechanism by which PUAs induced those differences remains uncertain. Moreover, to our knowledge, there is no data describing what developmental processes and germ layers are altered by PUAs.

In this work, we study if PUAs affect the embryonic development of the appendicularian urochordate specie *Oikopleura dioica* for two main reasons. First, from an ecological perspective, appendicularians (a.k.a. larvaceans) are relevant because they are cosmopolitan pelagic filter-feeding organisms, considered the second most abundant after copepods in marine mesozooplankton^25,26^. Appendicularians graze about 10% of the ocean’s primary production^27^. The appendicularian capability of trapping a wide range of particle sizes thanks to their unique filter-feeding apparatus house makes them to occupy an important trophic position in food webs. Appendicularians act as an important short-circuit that allows a rapid energy transfer from colloidal carbon and phytoplankton primary producers to zooplanktivorous predators such as fish larvae^28,29^, contributing at the same time to at least 8% of the vertical carbon transport to the deep ocean^30–32^. Therefore, the study of PUAs toxicity on the biology of appendicularians is fundamental from an ecological perspective to better understand the potential effect of increased amount of oxylipins on marine food webs and carbon cycle, upon potential intensification of harmful diatom blooms in the context of ocean warming and acidification.

Second, from an evolutionary and developmental biology (evo-devo) perspective, the chordate *O. dioica* is an attractive animal model because it has undergone a process of genetic and morphological simplification during the evolution of urochordates^33–36^. The low genetic redundancy of *O. dioica* genome, in comparison with the twice-duplicated vertebrate genome (reviewed in the ref. ^37^), together with the extraordinary amount of gene losses^36^, make that its functional redundancy is lower than in other animal species (e.g., vertebrates). Some gene losses have led this organism to be considered as an evolutionary knockout that can facilitate the dissection of complex genetic networks^36,38–41^. *O. dioica* has lost, for instance, the retinaldehyde dehydrogenase *Aldh1a* and most of the components of the metabolic pathway of retinoic acid (RA)^42^, a signaling molecule that in all other chordates plays a fundamental role in the regulation of embryo development, adult organ homeostasis and gametogenesis^43–45^. The loss of the *Aldh1a* in *O. dioica* is particularly relevant for the present work because it has been proposed that PUAs such as *trans,trans*-2,4-decadienal (DD) can compete with retinaldehyde for the substrate binding site of the Aldh1a^46^. This finding suggested the hypothesis that the teratogenicity of DD could be due to its interference with RA-synthesis, impairing thereby normal RA signaling during embryo development. Thus, our work aims to study *O. dioica* as an *Aldh1a* evolutionary knockout to test this hypothesis, because DD should produce minor or no alterations on *O. dioica* embryo development if RA signaling is the main developmental pathway targeted by DD. On the contrary, if DD severely affects the development of *O. dioica*, it would imply that other developmental mechanisms, distinct from RA signaling, are affected by DD exposure.

This work reveals that oxylipins can induce aberrant development and gene expression of appendicularians, even at concentrations in the same range than those measured after blooms, and puts an alert on the potential impact that an increase of biotoxins may have on marine food webs.

## Results

### Dose-dependent effects of DD on embryo development of *O. dioica*

**Fig. 1:**
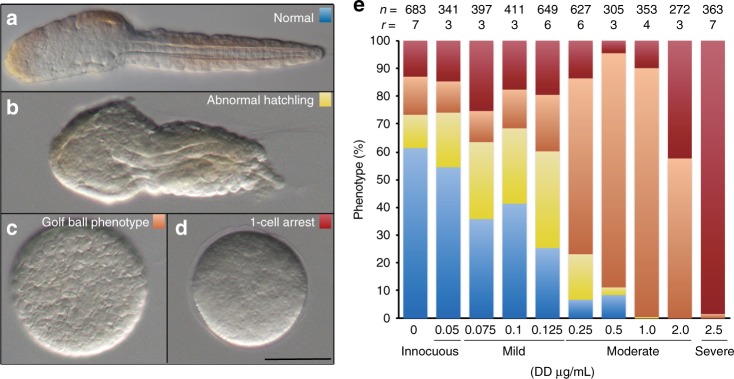
Effects and dose-response of DD on Oikopleura dioica embryo development. After 4.5 h of DD treatment, embryo phenotypes in A-D could be classified in four categories: **a** normal hatchling (blue), **b** aberrant hatchling (yellow), **c** Pre-tailbud arrest with a golf ball phenotype (salmon), and **d** 1-cell arrest (cherry). Scale bar 50 µm. **e** The proportion of each aberrant phenotype at different DD concentrations allowed us to characterize the dose response and to define four ranges of concentrations depending on their severity: innocuous, mild, moderate and severe. Statistical analyses and individual data plotting are provided in Supplementary Fig. 1. Colors correspond to phenotypes in **a**. Number of analyzed embryos (n) and number replicates (r) are indicated on top of each treatment

To investigate the possible teratogenic effects and dose-response of DD on *O. dioica* embryo development, we analyzed, at the time of hatching, embryos derived from eggs treated with DD at different concentrations (from 0.05 to 2.5 µg mL^−1^, this is from 0.33 to 16.42 µM). In contrast to DMSO-control conditions, DD-treatments altered embryo development in a dose-dependent manner, in which the severity of the phenotypic aberrations and the proportion of affected embryos depended on the concentration of the DD (Fig. 1). We classified the aberrant phenotypes in three categories: abnormal hatchlings, pre-tailbud arrested embryos with golf ball morphology, and 1-cell arrested zygotes (Fig. 1a–d). According to the differences in the proportions of aberrant phenotypes (statistical analyses are provided in Supplementary Fig. 1), we classified DD concentrations in four categories (Fig. 1e): innocuous (≤0.05 μg mL^−1^), in which no significant differences were observed in comparison with DMSO-controls (*P*-value 0.998); mild (from 0.075 to 0.125 μg mL^−1^), in which most embryos did hatch, but the presence of aberrant morphologies with shorten or kinked tails was higher than in the DMSO-control condition; moderate (from 0.25 to 2.0 μg mL^−1^), in which most animals did not hatch, remaining arrested in a pre-tailbud stage with the appearance of a golf ball; severe (≥2.5 μg mL^−1^), in which most zygotes remained at 1-cell stage with no cleaving.

### One-cell arrest embryos by severe DD concentrations

**Fig. 2:**
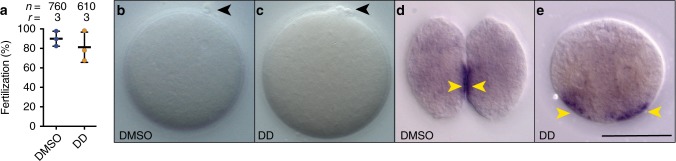
Characterization of 1-cell arrest phenotype caused by severe DD concentrations (2.5 µg mL^−1^). **a** Percentage of fertilized embryos assessed by the formation of polar bodies do not show significant differences (*P*-value = 0.432) between DMSO-control (blue circles in the plot) (**b**) and DD-treated embryos (orange circles in the plot) (**c**), in which moreover no morphological differences in the presence of internal granules nor surface rugosity are observed. Maternal *Wnt11* signal appeared asymmetrically distributed, mostly accumulated near the cell membrane (yellow arrowheads) that will form the prospective posterior vegetal pole both in DMSO-control (**d**) and DD-treated embryos despite the absence of the first cleavage (**e**), suggesting that DD does not alter the cellular architecture of the oocyte that drives primary axial polarity. Number of analyzed embryos (n) and number replicates (r) are indicated on top of each treatment. Reported means and standard deviations are represented in the plot with horizontal line and error bars, respectively. Scale bar 50 µm

To test if the absence of cell divisions at severe concentrations could be due to fertilization impairment, we scored for the formation of polar bodies after fertilization in the presence or absence of DD in 760 and 610 embryos, respectively (Fig. 2a–c). Results showed no significant difference in the number of oocytes with polar bodies between DMSO-controls and DD-treatments (ANOVA test, *P*-value = 0.432), suggesting therefore that DD was not impairing oocyte fertilization. Careful inspection of oocytes by differential interference contrast (DIC) microscopy did not reveal any evident morphological difference, neither in the shape and size of the internal granules of the oocytes, nor in the changes of membrane surface rugosity that normally occurs during fertilization (Fig. 2b, c). To investigate if the internal organization of the cytoplasm of oocytes could be affected by DD, we performed whole-mount in situ hybridizations to detect maternal *Wnt11* transcripts (Fig. 2d, e). In DMSO-control two-cell stage embryos at 30-min post-fertilization (mpf), maternal *Wnt11* signal appeared asymmetrically distributed, mostly accumulated near the cell membrane that will form the prospective posterior vegetal pole of the embryo (yellow arrowheads in Fig. 2d, and unpublished data). In DD treated embryos at 30-mpf, maternal *Wnt11* signal also appeared asymmetrically distributed near the membranes of the putative posterior vegetal pole, despite the absence of embryo cleavage, suggesting no changes of cellular architecture that drives the primary axial polarity (Fig. 2e).

### Golf ball pre-tailbud arrested embryos

**Fig. 3:**
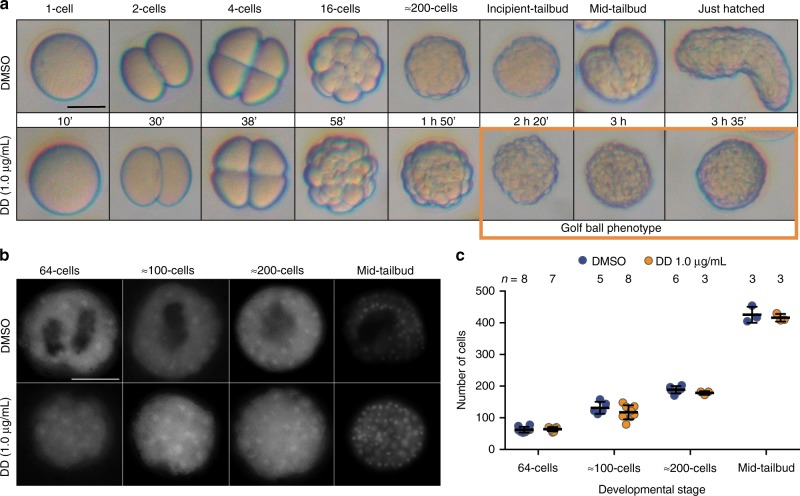
Characterization of the golf ball morphology at the pre-tailbud arrest stage caused by moderate DD concentrations (1 µg mL^−1^). **a** Developmental progression of DD-treated embryos does not show any obvious abnormality until 3 hpf, when morphogenesis starts during the formation of tailbud stages in DMSO-control embryos, but a golf ball morphology appears in an arrested pre-tailbud stage of DD-treated embryos (orange square), in which no trunk nor tail can be distinguished. **b** Nuclear staining with HOECHST reveals no significant differences in the number of cells between DMSO-control and DD-treated embryos at 64-cell, 100-cell, 200-cell, and mid-tailbud stage (ns, *P*-values 0.725, 0.262, 0.206, respectively). Images in **b** correspond to embryos in which *Bra* expression had been detected by whole-mount in situ hybridization, to test whether cell division had not slowed down not even in embryos that showed delay in the expression onset as described in Fig. 4. (**c**), suggesting that DD does not induce a general slow-down or blockage of development by the pre-tailbud arrest that produces the golf ball morphology. Reported means and standard deviations are represented in the plot with horizontal line and error bars, respectively. Scale bars 50 µm

At moderate DD concentrations (e.g., 1 μg mL^−1^), the majority of embryos proceeded normally with embryo cleavage, but most of them never developed a normal tailbud morphology (Figs. 1c, 3a). Comparison between the development of DMSO-control and DD-treated embryos revealed no obvious differences up to the incipient-tailbud stage about 3 h post-fertilization (hpf). At that stage, however, while control embryos showed a clear indentation at the initial demarcation of the trunk and tail, DD-treated embryos did not show that indentation, and their morphology looked like a golf ball (Fig. 3a). During tailbud stages, while control embryos continued with the progressive differentiation of their trunk and tail until the hatch, DD-treated embryos maintained the golf ball morphology in an apparently arrested pre-tailbud stage, in which no trunk nor tail could be distinguished (Figs. 1c, 3a).

To test if the failure to proceed to tailbud stages was due to the arrest of cell divisions, we counted the number of nuclei in control and DD-treated embryos, (Fig. 3b), we found no significant differences (*P*-values > 0.05, Fig. 3c), suggesting that the absence of trunk and tail differentiation, and the lack of obvious internal structures were not due to a slow-down or blockage of development caused by a deceleration or arrest of nuclear divisions, but to the failure of the process of morphogenesis that normally start at incipient-tailbud stage.

### DD causes zygotic expression delay of developmental genes

**Fig. 4:**
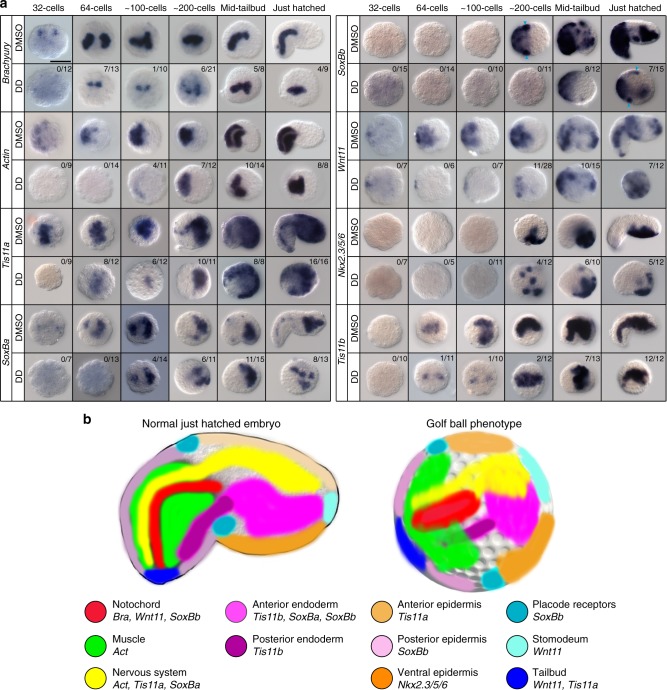
Alteration of developmental gene expression patterns by DD causing the golf ball morphology. **a** Whole mount in situ hybridizations on DMSO-control embryos and DD-treated embryos at 1 µg mL^−1^ was performed throughout different developmental stages until hatch (i.e., 32-cell, 64-cell, 100-cell, 200-cell, mid-tailbud stage and just hatched) using markers for derivatives of all three germ cell layers: mesoderm (notochord labeled by *Bra, Wnt11* and *SoxBb*, and muscle labeled by *Act*), endoderm (entire endoderm labeled by *Tis11b*, only endoderm of the trunk labeled by *SoxBa* and *SoxBb*), and ectoderm (epidermis of the tail labeled by *SoxBb*, epidermis of the trunk labeled by *Tis11a*, and ventral epidermis of the trunk by *Nkx2.3/5/6*; neural cells by *Tis11a* and *SoxBa*, and presumptive placodal regions by *Wnt11* and *SoxBb*; blue arrowheads label precursor cell of Lagerhand receptors, visible from a dorsal view). Numbers of scored embryos with signal are shown in the top-right corner of each panel. The rest of the scored embryos showed no signals. All DMSO-control embryos presented signal as shown. When possible, lateral views of embryos have been oriented anterior and dorsal to right and top, respectively. Scale bar 50 µm. **b** Colored schematic representation of the developmental gene expression domains in embryos with normal or golf ball phenotype as shown in **a**. Despite the failure to begin the process of morphogenesis of DD-treated embryos with golf ball morphology, at the time in which DMSO-control embryos have hatched, developmental gene markers show that the initial cell fate determination of derivatives of all three germ cell layers is not affected, and embryos show a correct AP and DV axial patterning. The main developmental processes that seem to be mostly affected are the failure of the formation of the indentation that separates trunk and tail, a delayed midline convergence, and the absence of tail elongation

The fact that *O. dioica* has lost RA signaling^42^ allowed us to discard that these abnormalities were due to alterations of this signaling pathway. To uncover what genetic mechanisms could be affected by DD resulting in a failure of morphogenesis and organogenesis, we performed expression analyses by whole-mount in situ hybridization with eight specific gene markers across different embryonic germ layers comparing six developmental stages between DMSO-control and DD-treated embryos (Fig. 4a). With the exception of *Brachyury* (*Bra*)^47,48^, *Actin* (*Act*)^49^, and *Nkx2.3/5/6* (ID comp20628 in^50^), all other *O. dioica* genes used in this work have not been described yet, and here they have been used as makers of derivatives of the three germ cell layers (Fig. 4b).

Expression analyses by whole-mount in situ hybridization revealed that the main alteration produced by DD exposure was that most embryos showed a systematic delay in the expression onset of all developmental genes analyzed. For instance, the first signal corresponding to the expression onset of *Bra*, *Act, Tis11a*, and *SoxBa* was delayed from 32-cell to at least 64-cell stage, the zygotic expression onset of *Wnt11* was delayed from 64-cell to at least to 200-cell stage, and the expression onset of *SoxBb* was delayed from 200-cell stage to an embryo with a golf ball appearance equivalent to a mid-tailbud stage in the control condition (Fig. 4a). This delay was maintained during development since expression domains at later developmental stages in DD-treated embryos reminded those at earlier stages of control embryos (Fig. 4a). The fact that the total number of cells at each stage did not significantly differ between control and DD-treated embryos (Fig. 3b, c) suggested that the delay of expression could not be explained by an overall developmental slowdown due to a decrease of the rate of cell divisions, but rather to alterations in the regulatory mechanisms of the transcription of these genes.

When we scored the number of DD-treated embryos that showed or not expression signal in each stage, we found that the delay of the expression onset could reach extreme situations of detecting no signal at all in a majority of DD-treated embryos at stages in which gene expression was already detected in 100% of their control counterparts (Fig. 4a). Remarkably, while the proportion of DD-treated embryos with no expression between 32-cell and 200-cell stages was high (73%, 210 out of 288), this proportion decreased in golf ball embryos equivalent to tailbud stages (31%, 58 out of 190). To determine whether the absence of signal was due to RNA degradation or repression of zygotic transcription, we focused on the expression signal of *Wnt11* gene, which has both maternal and zygotic components. Results revealed that while signal from the maternal component was observed up to the 64-cell stage in control embryos, time at which zygotic transcription started, in DD-treated embryos, however, only signal from the maternal component was observed and no signal from zygotic expression of *Wnt11* was detected until the 200-cell stage (Fig. 4a). This finding suggested that the absence of expression signal in DD-treated embryos was not due to transcript degradation, but it was consistent with a considerable delay and silencing of zygotic transcription of developmental genes, which was more conspicuous at earlier stages up to 200-cell stage than at later stages equivalent to tailbud in control embryos.

### DD alters midline convergence and posterior elongation

To test if the delay of the expression of developmental genes caused by DD was also accompanied by a delay of the onset of cell invagination during gastrulation, we analyzed *Bra* expression, which in agreement with previous reports^47^ labeled one pair of blastomeres (A21) at the surface in embryos at 32-cell stage, and two pair of cells that already occupied an internal position (A211 and A212) after invagination as gastrulation proceeded at 64-cell stage in control embryos (Fig. 4a). In DD-treated embryos, however, no signal was detected at 32-cells stage, and the first *Bra* signal was observed in only one pair of internal cells rather than two pairs as expected at the 64-cell stage (Fig. 4a). The fact the first two cells expressing *Bra* were already located in an internal position of the embryo (rather than the surface) suggested that DD did not delay the process of gastrulation, but simply altered the time at which *Bra* started to be expressed.

Analyses of the temporal dynamics of markers such *Bra* and *Act* revealed that the process of midline convergence seemed to be clearly delayed in DD-treated embryos. For instance, while the processes of notochord intercalation and muscle midline bilateral-convergence were completed by 2 hpf at 200-cell stage in control embryos (Fig. 4a), in DD-treated embryos these processes were not completed up to 2 h later by the time at which normal animals were already hatching (Fig. 4b). Moreover, the length of the *Bra* and *Act* expression domains appeared to be much shorter in DD-treated embryos than control ones, suggesting that not only the midline convergence was affected, but also posterior elongation was impaired.

### DD alters morphogenesis but no axial patterning or cell fate

Analysis of expression of eight developmental markers in DD-treated embryos with golf ball phenotypes revealed that the setup of the embryonic axes had been already established, and the fate determination of tissues derived from the three germ layers did not appear to be affected. For instance, the setup of the anterior-posterior (AP) axis in embryos with golf ball morphology was revealed by the presence of *Bra, Act*, and *SoxBb* expression domains restricted to the presumptive posterior half of the embryo, (labeling the mesodermal precursor of the notochord, muscle cells and the ectoderm of the tail, respectively, Fig. 4a, b), as well as *SoxBa*, *SoxBb*, *Tis11a*, and *Nkx2.3/5/6* expression domains restricted to the presumptive anterior half of the embryo (labeling the endoderm, and the ectoderm of the trunk Fig. 4a, b). The setup of the dorso-ventral (DV) axis was revealed by the restricted expression of *Nkx2.3/5/6* to the presumptive ventral epidermal domain of the trunk (Fig. 4a, b). In addition to the endodermal, mesodermal and ectodermal precursors labeled with the aforementioned markers in DD-treated embryos, the presence of ectodermal *SoxBb* and *Wnt11* expression domains in precursor cells of the Langerhans receptors in the tail-trunk transition area and in the most anterior part of the embryo (Fig. 4a, b) suggested that the cell fate commitment of placode and neural cell precursors was not affected by DD. Thus, despite that DD seems to block morphogenesis and the formation of the trunk and tail in embryos with a golf ball phenotype, the setup of the embryonic axes, as well as the fate determination of tissues derived from the three germ layers, seemed not to be altered by DD.

### Developmental genetic response of the defensome against DD

**Fig. 5:**
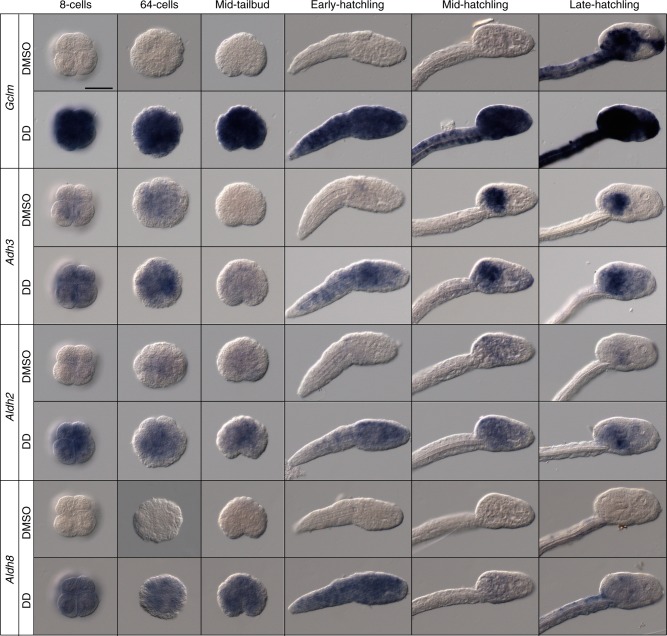
Defensome gene response to DD during embryo development. Whole mount in situ hybridizations on DMSO-control embryos and DD-treated embryos at 0.1 µg mL^−1^ was performed throughout different developmental stages until hatch (i.e., 8-cell, 64-cell, and mid-tailbud stages and early-, mid- and late-hatchlings). Expression analysis of *Gclm, Adh3*, *Aldh2*, and *Aldh8* reveals that DD activates a rapid response of the defensome during *O. dioica* development. The number of scored embryos in each stage after DD treatment was between 5 and 10, and in 100% of the cases showed the same expression pattern. Scale bar 50 µm

Previous works on sea urchins had revealed the existence of a defensome, a set of stress response genes that activates their expression during development to protect embryos upon environmental stress^20^. To test if DD upregulates defensome genes during *O. dioica* development, we have analyzed the expression pattern of four defensome genes—three previously described dehydrogenase genes *Adh3*^51^, *Aldh2*^52^, and *Aldh8*^42^, and a here newly described glutathione-related gene (Glutamate-cysteine ligase modifier subunit; *Gclm*)—in six developmental stages from eight-cell stage to late hatchling of DMSO-control and DD-treated embryos at mild concentrations (e.g., 0.1 µg mL^−1^) (Fig. 5). In control embryos, the expression signal of all four genes was almost negligible at early developmental stages, and it was only detected in mid/late hatchling stages, in which *Gclm* and *Adh3* showed obvious expression domains restricted to digestive compartments (e.g., mouth, pharynx, esophagus, and stomach lobes), and *Aldh2* and *Aldh8* showed either no signal or a weak and scattered signal (Fig. 5). In DD-treated embryos, however, the expression of all four genes was upregulated since very early developmental stages, showing broader and stronger staining than control embryos (Fig. 5). The obvious up-regulation of these four genes in response to DD, therefore, suggested that DD was able to trigger a genetic response of members of the defensome involved in aldehyde detoxification, and especially members that respond to alterations of the metabolism of the glutathione system such as *Gclm*, which was the gene that showed a stronger level of upregulation in response to DD (Fig. 5). Moreover, the fact that the expression of defensome genes was already noticeably upregulated by eight-cell stage, a time at which normal zygotic expression of most genes had not started yet, suggested that these genes had an immediate response capability upon DD challenge (Fig. 5).

### Effects of diatom extracts on *O. dioica* embryogenesis

To test if similar developmental abnormalities induced by DD on *O. dioica* could be also caused by natural PUAs or other oxylipins produced by diatoms frequently involved in algal blooms, we tested the effect of the extracts from different microalga species on *O. dioica* embryogenesis. These species included *Skeletonema marinoi (Sm)*, which has been characterized by its high content of the PUAs heptadienal and octadienal and the lack of decadienal^5,53^; *Chaetoceros affinis* (*Ca*), which produces other oxylipins different from PUAs^8^; *Prorocentrum minimum* (*Pm*), a dinoflagellate species used as a negative control that does not produce oxylipins^8^; and *Chaetoceros calcitrans* (*Cc*), a diatom included in the diet used to feed *O. dioica* in our animal facility^41^, but for which the capability to produce PUAs was unknown. First, we studied the dose-response of *O. dioica* development at different concentrations of *S. marinoi* extracts (from 5 to 100 μg mL^−1^). Results revealed that the most concentrated extracts reproduced the same phenotypes observed during DD treatments (Supplementary Fig. 2). Our results from treatments with *S. marinoi* extracts at 100 μg mL^−1^—which according to previous studies^53^ contained PUAs at a concentration of approximately 2.4 μg mL^−1^ (20.3 μM assuming an average MW of 117.17 for heptadienal and octadienal most abundant PUAs described in *S. marinoi*)—revealed that the golf ball morphology of pre-tailbud arrested embryos was the most abundant phenotype, suggesting that the effects of the PUAs in 100 μg mL^−1^ *S. marinoi* PUAs were comparable with the moderate, but not severe, condition observed in DD-treatments (Fig. 1 and Supplementary Fig. 2). These results were consistent with previous observations showing that higher concentrations of heptadienal and octadienal, the main PUAs of *S. marinoi*, were required to cause similar teratogenic effects to those of DD during sea urchin embryonic development^12^.

**Fig. 6:**
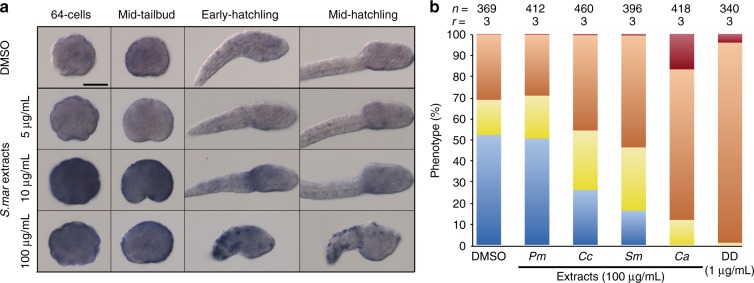
Effects and defensome response of *Oikopleura dioica* embryos treated with extracts of different diatom species. **a** Whole mount in situ hybridizations with *Gclm* on DMSO-control embryos and embryos treated with extracts of *S. marinoi* at different concentrations suggest that other PUAs than DD (e.g., heptadienal and octadienal) also activates a quick response of the defensome system. Scale bar 50 µm. **b** Embryo phenotypes observed after treatments with extracts of *S. marinoi* (Sm), *C. affinis* (Ca), *C. calcitrans* (Cc) corresponds to the same observed after DD-treatment (Fig. 1). Extract of *P. minimum* (Pm), a dinoflagellate that does not produce aldehydes, was used as additional control. Statistical analyses and individual data plotting are provided in Supplementary Fig. 2

To test whether treatments with *S. marinoi* extracts were also capable to trigger a defensome response as observed with DD, we performed in situ hybridization experiments with *Gclm*. Results revealed that *Gclm* expression was upregulated even after treatments with extracts at concentrations of only 10 μg mL^−1^, in which no significant difference was observed in the proportion of aberrant phenotypes between Sm-extract treated embryos and the DMSO-control condition (*P*-value = 0.261; Fig. 6a and Supplementary Fig. 2). We concluded therefore that potential secondary metabolites produced by *S. marinoi* were also capable to activate the expression of the defensome.

Finally, we tested whether *O. dioica* development was also affected by extracts of other microalgae that produce other secondary metabolites different from those of *S. marinoi*, using diatom extracts of *C. affinis* and *C. calcitrans*, as well as the dinoflagellate *P. minimum* as negative control. In all cases, with the exception of the negative control *P. minimum*, extracts affected *O. dioica* development giving rise to the same phenotypes as observed with DD and *S. marinoi* extracts (Fig. 6b and Supplementary Fig. 2). Results revealed that the proportion of normal embryos in *C. affinis* extract treatments seemed even lower than those observed with *S. marinoi* extracts, suggesting that *O. dioica* development could be even more sensitive to the toxicity of other oxylipins different than PUAs (Fig. 6b). In the case of *C. calcitrans*, a diatom whose production of PUAs or other oxylipins had not been investigated to our knowledge, we observed that embryo development was similarly affected by treatments with *S. marinoi* extracts (Fig. 6b). To our knowledge this is the first finding suggesting that *C. calcitrans* is able to produce toxic secondary metabolites, which indeed is consistent with personal observations during the feeding strategy in our *O. dioica* facility in which excess of this alga in the diet made *O. dioica* cultures to decline^41^.

## Discussion

Understanding the potential impact that global climate change may have on oceans’ health, and particularly how the potential increase of harmful algal blooms may impact marine trophic webs is one of the main challenges that our Society is currently facing^1^ (see focus areas and agenda of UNESCO-IOC). In this context, results from this work reveal that biotoxins from the oxylipin family that are abundantly released at the end of diatom blooms can compromise embryonic development of appendicularian species, and therefore their reproduction. This finding is ecologically relevant because appendicularians together with copepods, which are also affected by these biotoxins^4^, represent the two most abundant components of the mesozooplankton around the globe, playing a crucial role in the global dynamics of marine food webs^25^. Our analysis of the development of *O. dioica* embryos exposed to DD, which is the most investigated PUA among those synthesized by some bloom-forming diatoms species^3,54^, reveals a characteristic set of embryonic aberrant morphologies with frequencies that follow a defined dose-response pattern (Fig. 1). Our work, furthermore, reveals that the same set of embryonic aberrant morphologies is also induced by treatments with extracts from diatom species that produce high levels of heptadienal and octadienal (*S. marinoi*^5,53^), or other oxylipins different from PUAs (*C. affinis*^8^). Also extracts from species such as *C. calcitrans*, whose oxylipin content is still unknown, affect *O. dioica* embryogenesis.

At present, our understanding of how PUAs toxicity released after blooms affect marine organisms in their natural habitats remains uncertain. Previous studies have quantified dissolved PUAs after blooms events in a range from 7.61 × 10^−6^ to 0.02 µg mL^−1^^55^, questioning laboratory experiments in which the concentrations of PUAs are of an order of magnitude higher than those described in the field^5,54^. Although in our study the most dramatic embryonic malformations mostly appear at concentrations of orders of magnitude higher than in the field (i.e. one-cell arrest at > 2.5 µg mL^−1^ or golf ball morphology at >0.25 µg mL^−1^), we want to remark that we observe lethal embryonic abnormalities at concentrations as low as 0.075 µg mL^−1^, which is in the same order of the maximum value measured in the field after diatom blooms (e.g., 0.02 µg mL^−1^ after a bloom of *S. marinoi* in the northwestern Adriatic Sea^55^). Thus, despite we do not see apparent abnormalities in embryos exposed during 4hpf to DD at concentrations lower than 0.05 µg mL^−1^, we cannot exclude that long or repeated exposure of appendicularians to peaks of PUAs released after blooms (see ref .^54,56^), or even synergistic effect of simultaneous exposure to multiple PUAs may have detrimental effects on appendicularians’ biology in natural habitats. This possibility is especially important considering the outstanding ability of appendicularians to concentrate microalgae. Moreover, our finding that longer exposures before fertilization, which facilitate eggs to accumulate DD through the chorion (Supplementary Fig. 3), increase the sensitivity of appendicularian development to DD toxicity makes plausible to suggest that adult animals exposed to DD throughout generations may accumulate this biotoxin in their gonads and become, therefore, more vulnerable to its detrimental effects. This possibility might explain the observation that overfeeding *O. dioica* cultures with the diatom *C. calcitrans* appears to lead culture declining^41^. Considering the ecological relevance of appendicularians in marine food webs and ocean carbon cycle, future experiments are urged to better understand the threat that oxylipins and other biotoxins can cause on the biology of appendicularians in natural environments. For instance, longer exposure regimes of *O. dioica* adults at low DD doses or overfeeding with excess of oxylipin-producing diatoms will be necessary to further investigate the vulnerability of appendicularians to the accumulation of these biotoxins throughout multiple generations.

Finally, our finding that *Gclm* and other defensome genes show highly sensitive expression responses to low concentrations of PUAs and other oxylipins (Figs. 5, 6) opens the possibility to use *Gclm* and other defensome markers as biosensors to test for genetic stress of natural appendicularian populations that may have been exposed to algal blooms in their habitats. Interestingly, increased expression of *Gclm* has been also reported in *Ciona intestinalis* larvae treated with DD^16^, suggesting that the use of *Gclm* expression as a biosensor can be used in other urochordates, and possibly other organisms, to monitor ecosystems with different exposures to blooms, thus providing a better understanding and a prediction of the potential impact of harmful algal blooms associated for instance to ocean warming and acidification.

Despite PUAs and other oxylipins produced by diatoms can interfere with a wide spectrum of physiological processes in many marine animals^9–18^, the genetic pathways altered by these biotoxins remain unclear, especially during embryo development. The fact that PUAs such as DD had been described to be able to compete with retinaldehyde for the substrate binding site of the Aldh1a enzymes had led us to hypothesize that genetic alterations due to a disruption of RA signaling could be a potential explanation for the physiological and developmental alterations caused by DD^46^. Although many studies have reported the teratogenic effect of DD on different marine organisms^11,16,57–61^, the direct effect of DD on RA signaling pathway has never been investigated in vivo, and whether changes detected by RT-PCR of the expression levels of some typical RA-target genes such *Hox1*, *Hox12*, and *Cdx* in ascidians was due to RA or some other signaling pathway remained unknown^15^. Our study, taking advantage that *O. dioica* is a natural evolutionary knockout of RA signaling^42^, demonstrates for the first time that DD can cause dramatic developmental alterations independently of RA signaling.

To our knowledge, this work is the first general attempt to fill the gap between the teratogenic phenotypes and the developmental genetic responses induced by oxylipins by expression analyses of whole-mount in situ hybridization. Our results show which developmental processes are altered and which ones are not affected by DD. Indeed, early developmental processes, such as cleaving pattern, gastrulation, and cell division rate do not seem to be affected by the exposure to moderate concentrations of this biotoxin. DD-treated embryos, however, fail to begin the process of morphogenesis, and develop a golf ball morphology in which the indentation that normally separates the trunk from the tail at incipient-tailbud stage never appears. Molecular characterization of DD-treated embryos reveals a systematic delay of the onset of the expression of developmental genes, completely abolishing in many cases zygotic expression until late developmental stages. The fact that defensome genes highly upregulate their expression as a quick response to the presence of DD, even as early as at the eight-cell stage, when most genes have not yet started normal zygotic transcription, indicates that downregulation of developmental genes does not result from a general transcriptional repression. On the contrary, it points to the activation of a silencing mechanism for developmental genes, especially during early stages, that could be part of the embryonic response to environmental challenges.

Despite the absence of morphogenesis, the expression of specific cell markers in golf ball reveals a correct cell fate differentiation of the main derivatives of the ectodermal, mesodermal and endodermal germ layers, as well a correct establishment of AP and DV axes that confer a correct spatial regionalization in DD-treated embryos (Fig. 4b). Analyses of the position and size of expression domains of developmental markers show that midline convergence and tail elongation are notably affected in DD-treated embryos, suggesting that genes regulating these two developmental processes may be linked to the formation of the golf ball morphology. Future transcriptomic profiling by RNAseq and functional approaches will be necessary to uncover the entire set of defensome genes that respond to DD exposure, as well as the developmental gene pathways altered by DD which are responsible of the failure of morphogenesis in *O. dioica*.

## Methods

### Animal culture and embryo collection

*Oikopleura dioica* embryos were obtained by in vitro fertilization from adults cultured in our animal facility at the University of Barcelona as described in the ref. ^41^. Briefly, animals were originally collected in the Mediterranean coast of Barcelona (Catalonia, Spain), and kept in the facility with a life cycle of five days at 19 °C. Animals are cultured in sterile seawater (sSW) filtered by 0.22 µm (VacuCap PF Filters 4622, Pall Corporation), and fed with four microalgae (*Isochrysis sp*., *C. calcitrans*, *R. reticulata* and *Synechococcus sp*). Mature females and males were collected at day 5, and after natural spawning, eggs were in vitro fertilized, and raised in petri dishes in sterile sea water until the desired stage.

### Decadienal treatments

Previous studies about the effect of *trans,trans*-2,4-decadienal (DD, Sigma-Aldrich®, W313505) on ascidians embryos had shown that the chorion could act as a barrier that slow down the access of DD to embryos^14,15^. Since our experience had shown that dechorionation is not possible in *O. dioica* embryos because it lethally affects development, to test if the chorion of *O. dioica* could also be a barrier against DD, we scored the hatching success of embryos that had been pre-incubated in DD previous to fertilization during different times (Supplementary Fig. 3). Thus, we empirically determined that despite the chorion of *O. dioica* also act as a barrier for exogenous DD, 10 min (min) of pre-incubation with DD before fertilization was enough to overcome the barrier. We therefore implemented a 10-min egg pre-incubation as the standard procedure in all our DD treatment experiments.

For DD treatments, pools of *O. dioica* eggs were incubated before fertilization and during development with nine increasing concentrations of *DD* 0.05, 0.075, 0.1, 0.125, 0.25, 0.5, 1.0, 2.0, and 2.5 µg mL^−1^ (0.33, 0.49, 0.66, 0.82, 1.64, 3.28, 6.57, 13.14, and 16.42 µM, respectively). Stock solution of 15 mg mL^−1^ of DD was prepared in dimethyl sulfoxide (DMSO), avoiding light exposure. To minimize the toxic effect of DMSO in the development of *O. dioica*, the stock solution was diluted 1:500 (30 µg mL^−1^; 0.2% DMSO) in sSW. This diluted stock was used to create each incubation solution containing the corresponding concentration of DD.

Eggs were pre-incubated for 10 min before fertilization into 900 µL of DD incubation solution. Then eggs were fertilized by adding 100 µL of sperm dilution (the sperm of two males diluted into 1 mL of sSW). Five minutes after the in vitro fertilization, fertilized eggs were transferred into a 50 mm-diameter-glass dish with 3 mL of the corresponding incubation solution. Control embryos were incubated in DMSO at 0.02%(v/v) without DD, which corresponded to the experimental condition at the maximum concentration of 2.5 µg mL^−1^ of DD. In experiments to obtain treated embryos for in situ hybridization, we added DMSO to have the same final concentration of 0.02%(v/v) in all experimental conditions. Embryos were cultured at 19 °C until hatching (≈4 hpf), time at which the effects of DD were scored. At least three replicates (r) containing hundreds of embryos (n) were analyzed for each condition.

### Expression analyses and nuclear staining

Developmental gene markers in *O. dioica* were identified by in silico screening of the Oikobase^62^, using *Ciona intestinalis* proteins as starting queries for tBLASTn searches against EST and genomic databases. The orthology of proteins was deduced by reciprocal best BLAST search against the *Ciona* genome (KH from Aniseed database). Whole-mount *in situ* hybridizations using probes of the genes of interest, which were generated by PCR amplification of cDNA with gene-specific primers (Supplementary Table 1), were performed as described in^42^. Embryos were fixed in 4% paraformaldehyde in fix buffer (0.1 M MOPS, 0.5 M NaCl, 2 mM MgSO_4_, 1 mM EGTA) for 1 h at room temperature, dehydrated in 70% ethanol and stored at −20 °C. Fixed embryos were dechorionated manually in petri dishes with poly-lysine as described in the ref. ^63^.

For nuclear staining, fixed whole embryos were rehydrated by washing in PBST during 10 min and were incubated in staining solution (1 µM Hoechst-33342 Invitrogen-62249 in PBST) for 5–10 min. ProLong Live Antifade Reagent (Invitrogen-P36975, dilution 1:100) was added to prevent photobleaching during fluorescence image capture. Nuclei were manually counted by analyzing the images of 10–15 optical sections for each embryo.

### Diatom extracts and treatments

Cultures of one liter of the diatoms *Skeletonema marinoi*, *Chaetoceros affinis*, *Chaetoceros calcitrans* and the dinoflagellate *Prorocentrum minimum* were grown up to stationary state to obtain extracts as described in the ref. ^53^ with minor modifications: microalgal pellets were frozen in liquid nitrogen and stored at −80 °C until cell lysis was performed by sonication. Stock solutions of 500 mg mL^−1^ of each extract were prepared in DMSO, avoiding light exposure. To minimize the toxic effect of DMSO in the embryo development of *O. dioica*, the stock solution was diluted 1:100 in sSW, which was used to create three incubation solutions at 5, 10, and 100 µg mL^−1^. Treatments were performed as described for DD. Considering the total number of cells of *S. marinoi* calculated from their concentration (i.e., 1 L at 1,650,000 cells mL^−1^) we perform an approximate calculation of how much PUAs could be present in the extract according to Barreiro et al.^53^, in which it has been reported that the total carbon content per cell is 20.7 pg C cell^−1^ and PUAs concentration is 8.5 µg PUAs mg C^−1^. Dry weight of total organic extracts were weighted in order that treatments could be referred in µg mL^−1^ (i.e., *S. marinoi* culture rendered 12.2 × 10^3^ µg of extract).

### Statistical analysis

Statistical analysis was performed using RStudio Version 0.98.507 for Mac OS X. The results were reported as means ± SD (standard deviation) and analyzed by Tukey HSD (honest significant difference) test for multiple comparisons between control and treatments and between treatments, and by ANOVA the pairwise comparisons. Prism GraphPad software (v. 7.0c) was used to generate plots showing the frequencies of the phenotypes in each replicate after treatments with DD and algal extracts.

## Electronic supplementary material

Supplementary Information

## Data Availability

All data generated or analyzed during this study are included in this published article and its supplementary information. The identification numbers of the sequences used to generate the probes for the in situ hybridization analyses listed in Supplementary Table 1 (GSOIDG00000279001, GSOIDG00000756001, GSOIDG00015222001, GSOIDG00010386001, GSOIDG00013526001, GSOIDG00011688001, GSOIDG00003812001, GSOIDG00017080001, GSOIDG00006303001, GSOIDG00000110001, GSOIDG00002220001, GSOIDG00021101001) correspond to the publicly available database Oikobase: https://www.oikoarrays.biology.uiowa.edu/Oiko/
