# Supplementary material for: Diatom bloom-derived biotoxins cause aberrant development and gene expression in the appendicularian chordate Oikopleura dioica
Source: Commun Biol. 2018 Aug 24;1:121. doi: 10.1038/s42003-018-0127-2 (PMC6123688; doi:10.1038/s42003-018-0127-2)
Supplement: Supplementary file 1 — Supplementary Information [file 42003_2018_127_MOESM1_ESM.pdf]

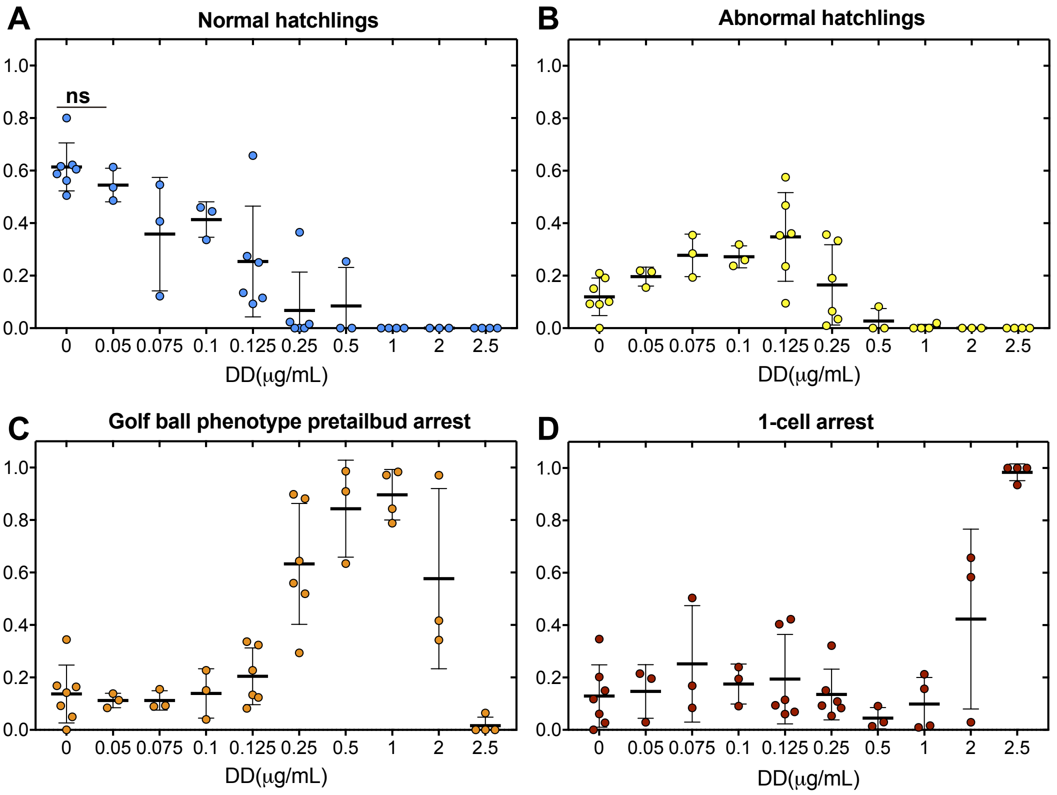

Supplementary Figure 1. Frequency of *Oikopleura dioica* embryo phenotypes after treatments with DD at different concentrations. **(A)** normal hatchling, **(B)** aberrant hatchling, **(C)** pre-tailbud arrest with a “golf-ball” phenotype, and **(D)** 1-cell arrest. For each condition, circles represent the frequency of the phenotype in each replicate individually analyzed (see **Fig 1E**). Reported means and standard deviations are represented with horizontal line and error bars, respectively. DD concentration at 0.05 μg/mL was considered innocuous, since no significant difference was observed in the amount of normal hatchlings in comparison with the DMSO-control condition (ns, P-value 0.99843). At higher concentrations than 0.05 μg/mL we started to observe severe abnormalities that normally we do not observe in DMSO-control conditions (see **Fig 1B**; P-values 0.1522 and <0.001, for 0.075 and 0.125 μg/mL, respectively).

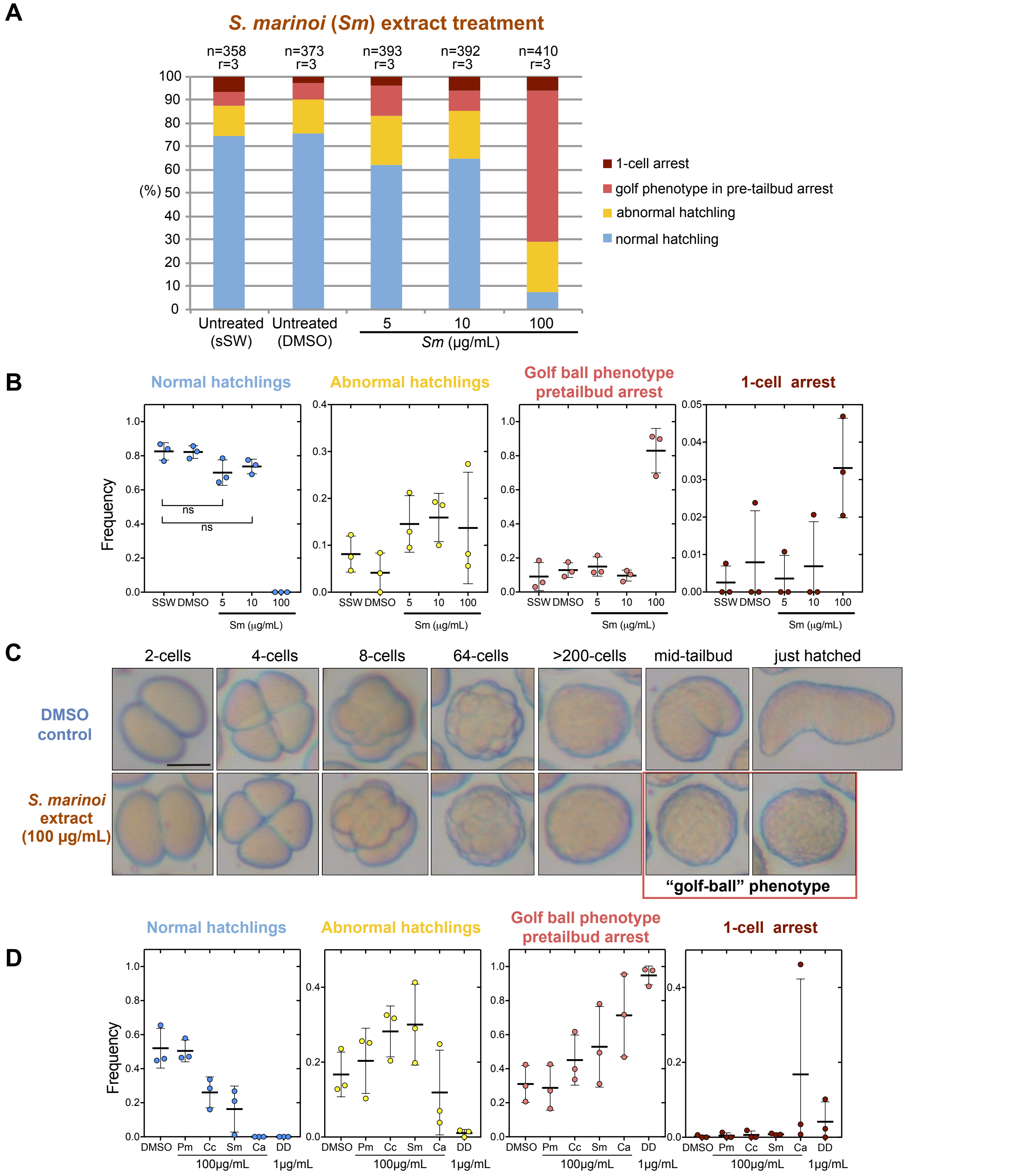

Supplementary Figure 2. Effects and dose-response of *Skeletonema marinoi* extracts on *Oikopleura dioica* embryo development. (A) After 4.5 hours of treatment with extracts of *S. marinoi* (*Sm*) at different concentrations (5, 10 and 100 µg/mL, which may correspond to PUAs approximate concentrations of 1, 2 and 20 µM, respectively), abnormal embryo phenotypes were the same as those obtained after DD-treatments (see **Fig 1**: normal hatchling (blue), aberrant hatchling (yellow), Pre-tailbud arrest with a golf ball phenotype (salmon), and 1-cell arrest (cherry)). The frequency of each abnormal phenotype depended on the extract concentration of the treatment. Number of analyzed embryos (n) and number replicates (r) are indicated on top of each treatment. **(B)** Plots of phenotype frequencies throughout treatments at different concentrations of *S. marinoi*. For each condition, circles represent the frequency of the phenotype in each replicate individually analyzed in (**A**). Reported means and standard deviations are represented with horizontal line and error bars, respectively. No significant differences were observed between embryos developed in sSW or DMSO (P-value 0.9996). Extracts at 5 and 10 µg/mL did not show significant differences with DMSO-control (ns, P-value 0.0573 and 0.2301 for normal hatchling phenotype, respectively). **(C)** The developmental progression of embryos treated with *S. marinoi* extract at 100 µg/mL reproduced the same alterations observed with DD at moderate concentrations (see **Fig 2**). Thus, no obvious abnormalities are observed until 3 hpf, time at which morphogenesis starts during the formation of tailbud stages in DMSO-control embryos, but a golf ball phenotype appears in arrested pre-tailbud stage of treated embryo. Scale bar 50 µm. **(D)** Plots of phenotype frequencies throughout treatments with different microalgal extracts at 100 µg/mL – *S. marinoi* (*Sm*), *C. affinis* (*Ca*), *C. calcitrans* (*Cc*) and *P. minimum* (*Pm*), and DD at 1 µg/mL. For each condition, circles represent the frequency of the phenotype in each replicate individually analyzed in **Fig 6B**. Reported means and standard deviations are represented with horizontal line and error bars, respectively.

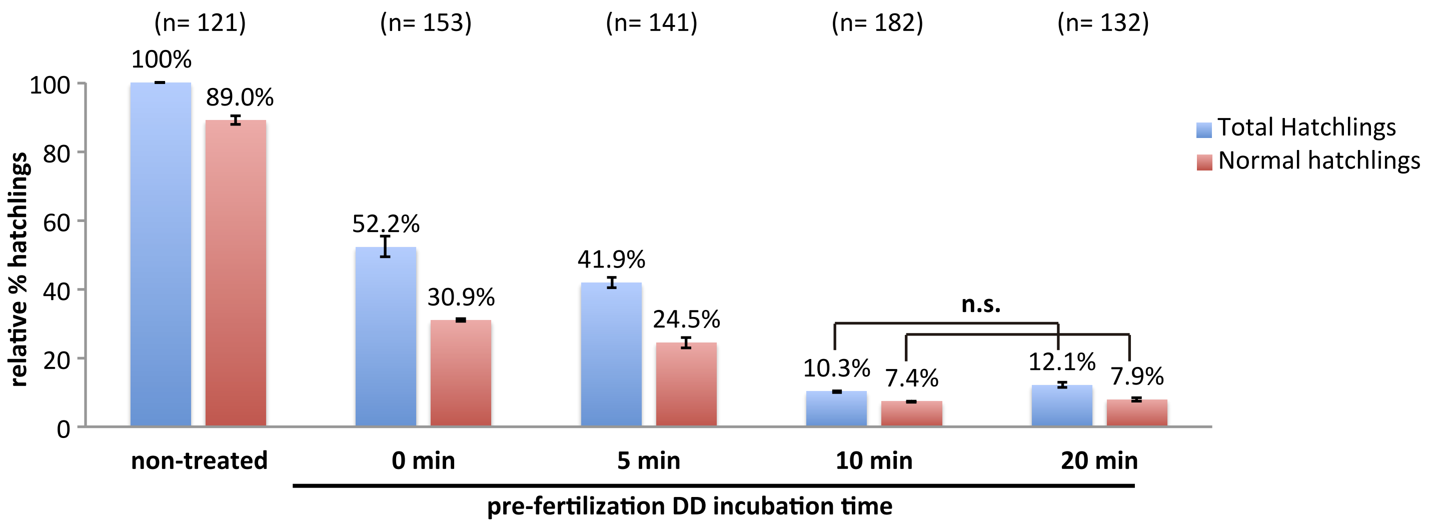

Supplementary Figure 3. Determination of the requirement of pre-incubation time of oocytes during DD treatments. Using a DD concentration of 0.28 *μg/mL* (1,84 *μM)*, oocytes were pre-incubated with DD during 0, 5, 10 and 20 minutes before fertilization, in at least 2 replicates, and at least 100 embryos analyzed (n) in each condition. After fertilization and continuous DD-treatment, we scored the percentage of animals that hatched (blue), and from those what proportion had a normal morphology (red). Error bars correspond to standard deviations. Results showed that the relative abundance of animals that hatched and those that had normal morphology in relation to non-treated embryos (negative control) was significantly lower when pre-incubations were shorter than 10 minutes. No significant (n.s.) decrease of hatch or normal morphologies was observed with longer pre-incubation times (P-values 1.0 and 0.99992, respectively). We therefore always used pre-incubation times of 10 minutes as the standard procedure in our treatment experiments.

Supplementary Table 1. Developmental and defensome gene markers. Oikobase IDs (http://oikoarrays.biology.uiowa.edu/Oiko), names and sequences.

Gene	Gene ID	Primer name	Sequence (5’- 3’)
Brachyury	GSOIDG00000279001	OdiT_F3	GGTTCGCACTGGATGAAACAGCC
		OdiT_R3	TATCCGTTCTGACACCAGTCGTTC
Actin	GSOIDG00000756001	Actin_5'_F	GTCCCCGCCATGTACGTCTG
		Actin_3'_R	GCATCGGAATCGCTCGTTACCA
Tis11a	GSOIDG00015222001	ZFC3Ha_e5_F	GGGTACTGCCCATATGGCG
		ZFC3Ha_e7_R	GCTCGAAGTTGGGCAGCTG
SoxBa	GSOIDG00010386001	SoxBa_e6_F	GCAGAAGTACCCAGCAAGGA
		SoxBa_e6_R	GTGACCACTTTCGGGCTTGT
SoxBb	GSOIDG00013526001	SoxBb_e4_F	GTTGTCGCTGGAAATGGCGA
		SoxBb_e8_R	CTCGACACGGACGCTCTGAT
Wnt11	GSOIDG00011688001	Wnt_s4_atgF	ATGAAGATTTCAGTCACCCTTTTCTCTG
		Wnt_s4_stopR	GTTATTTGCATATATGAGTGACAGTCG
Nkx2.3/5/6	GSOIDG00003812001	Nkx_5’start_F	GACCGAAAAATTACAACTATGAGC
		Nkx_ex_3_R	GCTGTAGCGCCGAGCTCAC
Tis11b	GSOIDG00017080001	ZFC3Hb_e2_F	GGCCAAATGAACGACGAAATCG
		ZFC3Hb_e4_R	GCACTCGGAGAGCGAGAG
Glcm	GSOIDG00006303001	Glcm_e2_F	GCAATAATTATCCAAGATGCCATG
		Glcm_e5_R	GTTCAGTCCTGCAAAGTATCC
Adh3	GSOIDG00000110001	Adh3_F1	CGTCGGTAAAGTGATCACGTGCA
		Adh3_R1	GCGCCCTGTGTAGCTCGGAC
Aldh2	GSOIDG00002220001	Aldh2_F	TGGAACTTCCCTCTCCTCATGCA
		Aldh2_R	TTATTTGGCGTATTGAGGAAGTTTCAT
Aldh8a1	GSOIDG00021101001	Aldh8_F	GATTTAAACAAAAAATGGAGCCGATTG
		Aldh8_R	TTTATTGCTTGCTAATTGTATTAGCTTGAAG
